# Double C–H bond activation of acetylene by atomic boron in forming aromatic cyclic-HBC_2_BH in solid neon†
†Electronic supplementary information (ESI) available. See DOI: 10.1039/c7sc01399j



**DOI:** 10.1039/c7sc01399j

**Published:** 2017-04-19

**Authors:** Jiwen Jian, Wei Li, Xuan Wu, Mingfei Zhou

**Affiliations:** a Department of Chemistry , Shanghai Key Laboratory of Molecular Catalysis and Innovative Materials , Collaborative Innovation Center of Chemistry for Energy Materials , Fudan University , Shanghai 200433 , China . Email: mfzhou@fudan.edu.cn

## Abstract

The organo-boron species formed from the reactions of boron atoms with acetylene in solid neon are investigated using matrix isolation infrared spectroscopy with isotopic substitutions as well as quantum chemical calculations. Besides the previously reported single C–H bond activation species, a cyclic-HBC_2_BH diboron species is formed *via* double C–H bond activation of acetylene. It is characterized to have a closed-shell singlet ground state with planar *D*_2h_ symmetry. Bonding analysis indicates that it is a doubly aromatic species involving two delocalized σ electrons and two delocalized π electrons. This finding reveals the very first example of double C–H bond activation of acetylene in forming new organo-boron compounds.

## Abstract 2

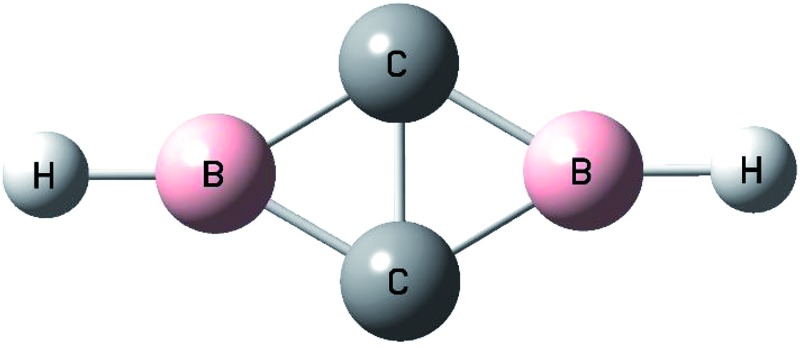
Boron atoms react with acetylene to form an aromatic cyclic-HBC_2_BH molecule *via* double C–H bond activation of acetylene in solid neon.

## Introduction

Carboranes, which are composed of boron, carbon and hydrogen atoms, are among the most studied organo-boron clusters due to their unique geometric and electronic structures and chemical reactivities.^1,2^ The activation of C–C and C–H bonds of hydrocarbon molecules by atomic boron in forming novel organo-boron compounds has been the subject of many experimental studies,^3^ which comprise gas phase kinetic^4,5^ and crossed molecular beam dynamic investigations,^6–8^ as well as matrix isolation spectroscopic studies.^9–13^ Experimental studies combined with theoretical calculations^3,14,15^ have provided detailed insight into the thermochemistry and mechanisms of the elementary reactions of atomic boron and hydrocarbons, which are also relevant to many chemical processes such as material synthesis, high temperature combustion and interstellar chemistry.^16^

The reaction of atomic boron with the simplest alkyne, C_2_H_2_, has received particular attention. The rate constants for this reaction have been measured in the temperature range of 23–295 K.^4^ It was found that the reactions have quite large rate constants.^4^ The results also suggest the presence of a small entrance barrier along the reaction path. The products from the reaction of pulsed laser-ablated boron atoms with C_2_H_2_ were studied by matrix isolation infrared absorption spectroscopy in a solid argon matrix.^10^ Three BC_2_H_2_ isomers, namely, the cyclic-B(C_2_H_2_) borirene radical, the inserted HBCCH molecule and the cyclic-HBC_2_H radical were identified. In addition, linear HBCC and HCCB species were also formed by the reaction of hyperthermal boron atoms and C_2_H_2_ during the sample deposition process.^10^ The reaction of ground state boron atoms with C_2_H_2_ was further studied under single collision conditions at different collisional energies using a crossed beam technique.^6^ Based on the collision-energy dependence of the differential cross-section and on the comparison of the experimental data with high level electronic structure calculations, the reaction was suggested to be dominated by an atomic boron *versus* atomic hydrogen replacement mechanism leading to the formation of two BC_2_H isomers: the linear isomer HBCC and the cyclic structure c-BC_2_H.^5^ Theoretical calculations suggested that the reaction proceeded with the initial formation of a cyclic-B(C_2_H_2_) borirene radical intermediate followed by hydrogen atom migration to form a cyclic HBC_2_H intermediate.^6^

Here we report a combined matrix isolation infrared spectroscopic and theoretical study on the reactions of boron atoms with acetylene in solid neon. We will show that besides the previously reported single C–H bond activation species, a cyclic-HBC_2_BH molecule is also formed *via* double C–H bond activation, which is characterized as having a closed-shell singlet ground state with planar *D*_2h_ symmetry that is doubly (σ and π) aromatic.

## Experimental details and computational methods

The experimental setups for pulsed laser evaporation and matrix isolation infrared spectroscopic investigation have been described in detail previously.^17^ The boron atoms were prepared by pulsed laser evaporation of a rotating bulk boron target using the 1064 nm fundamental of a Nd:YAG laser (Continuum, Minilite II, 10 Hz repetition rate). The evaporated boron atoms were co-deposited with acetylene reagent gas in excess neon onto a cryogenic CsI window maintained at 4 K by means of a closed-cycle helium refrigerator. The acetylene/Ne mixtures were prepared in a stainless steel vacuum line using standard manometric techniques. Natural abundance boron (^10^B, 19.8%; ^11^B, 80.2%) and ^10^B-enriched (97%) targets were used in different experiments. In general, after 30 min of sample deposition, the infrared spectra of the resulting samples were recorded in the transmission mode between 4000 and 450 cm^–1^ using a Bruker Vertex 80 V spectrometer at 0.5 cm^–1^ resolution. A liquid nitrogen cooled broad band HgCdTe (MCT) detector was used. Bare window backgrounds, recorded prior to sample deposition, were used as references in processing the sample spectra. After the infrared spectrum of the initial deposition had been recorded, the samples were warmed up to the desired temperature and quickly recooled and more spectra were taken. Broad-band photoexcitation was performed using a high-pressure mercury arc lamp with glass filters.

Quantum chemical calculations were performed to support the experimental assignments and to gain insight into the structure and bonding of the observed species. Geometry optimizations were carried out at the B3LYP^18^ and CCSD(T)^19^ levels of theory. The Dunning's correlation consistent basis set with polarized triple-zeta plus diffuse functions (aug-cc-pVTZ) was used.^20^ The stationary points were located without symmetry constraints with the Berny algorithm using redundant coordinates.^21^ Analytical Hessians were computed to determinate the nature of the stationary points and to describe the IR spectra.^22^ The harmonic vibrational frequencies were computed at both levels of theory. All calculations were performed using the Gaussian 09 program.^23^ Chemical bonding analyses were performed by the adaptive natural density partitioning (AdNDP) method^24^ using the density generated from the B3LYP calculations for the assignment of both localized and delocalized bonding using the Multiwfn program.^25^ Nucleus independent chemical shifts (NICS) were also computed at the gauge-including atomic orbital (GIAO) B3LYP/aug-cc-pVTZ level of theory.^26^ NICS values at the ring center (NICS(0)) and 1 Å above it (NICS(1)) were calculated. Due to the small size of the ring systems studied here, the NICS(1) values are used as the criterion to minimize the local shielding effects.^27^

## Results and discussion

**Fig. 1:**
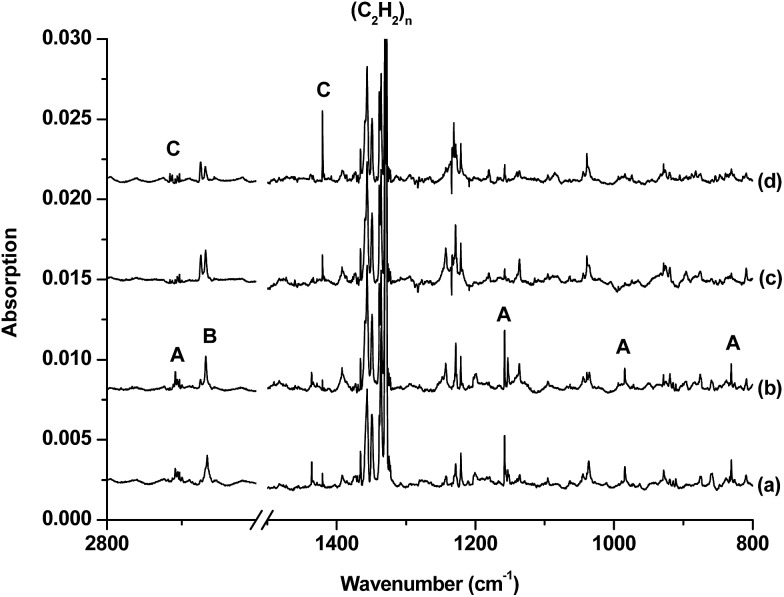
Infrared spectra in the 2800–2600 and 1500–800 cm^–1^ regions from co-deposition of ^10^B atoms with 0.05% acetylene in neon. (a) After 30 min of sample deposition at 4 K, (b) after annealing at 12 K, (c) after 15 min of *λ* > 280 nm UV light irradiation and (d) after 15 min of 250 < *λ* < 580 nm light irradiation.

**Fig. 2:**
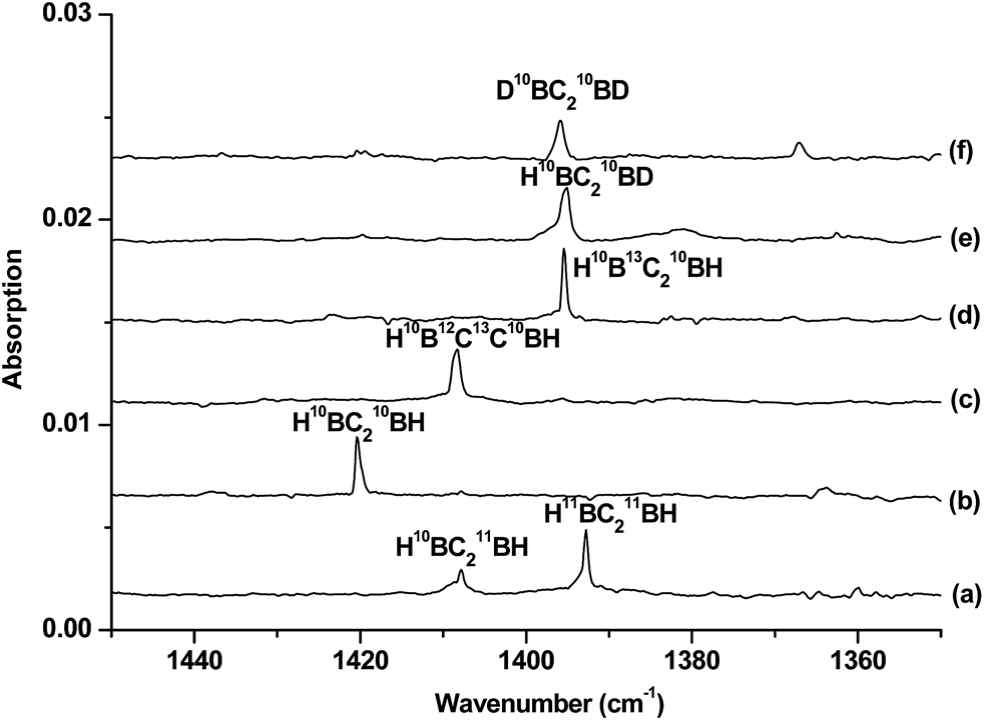
Difference infrared spectra in the 1450–1350 cm^–1^ region from co-deposition of boron atoms with isotopic-labelled acetylene in solid neon (the spectrum recorded after 15 min of 580 > *λ* > 250 nm light irradiation minus the spectrum recorded after annealing at 12 K): (a) natural abundance boron + 0.05% C_2_H_2_, (b) ^10^B + 0.05% C_2_H_2_, (c) ^10^B + 0.05% H^12^C^13^CH, (d) ^10^B + 0.05% ^13^C_2_H_2_, (e) ^10^B + 0.05% HCCD and (f) ^10^B + 0.05% DCCD.

**Fig. 3:**
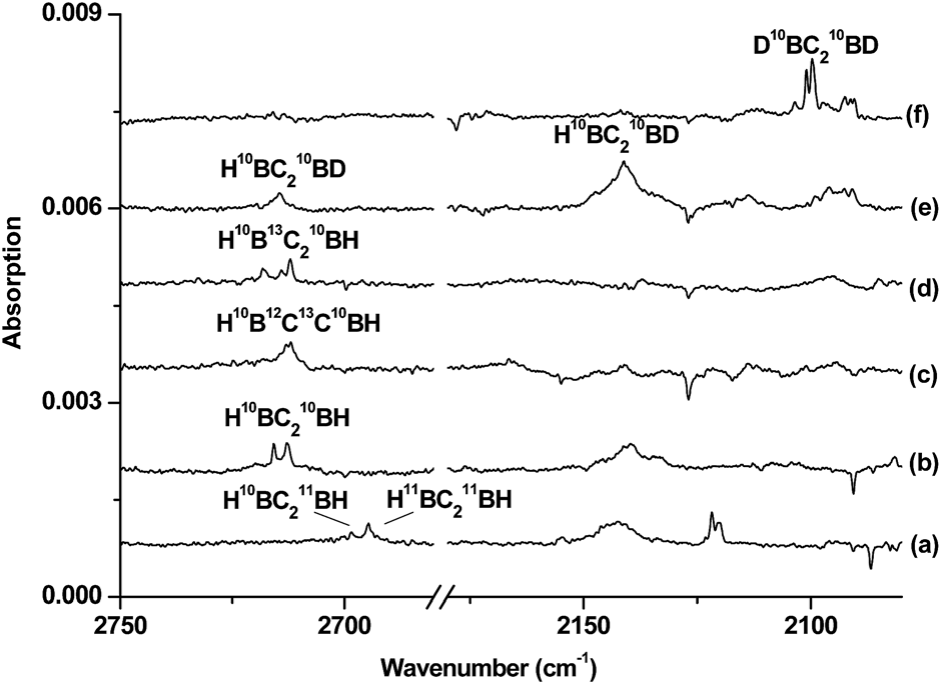
Difference infrared spectra in the 2750–2680 and 2180–2080 cm^–1^ regions from co-deposition of boron atoms with isotopic-labelled acetylene in solid neon (the spectrum recorded after 15 min of 580 > *λ* > 250 nm light irradiation minus the spectrum recorded after annealing at 12 K): (a) natural abundance boron + 0.05% C_2_H_2_, (b) ^10^B + 0.05% C_2_H_2_, (c) ^10^B + 0.05% H^12^C^13^CH, (d) ^10^B + 0.05% ^13^C_2_H_2_, (e) ^10^B + 0.05% HCCD and (f) ^10^B + 0.05% DCCD.

The infrared spectra in selected regions using a ^10^B-enriched target and 0.05% C_2_H_2_ are shown in Fig. 1. Besides the strong C_2_H_2_ and (C_2_H_2_)_*n*_ absorptions, product absorptions are observed either upon sample deposition or upon annealing or photolysis, which can be classified into several groups based on their annealing and photochemical behaviors (labeled as A–C in Fig. 1). Group A involves four absorptions with band centers at 2708.5, 1158.2, 984.3 and 831.0 cm^–1^. These absorptions are presented upon sample deposition, and increase together upon sample annealing at 12 K, but are almost destroyed under subsequent broad-band irradiation in the wavelength range of 280–580 nm using a high-pressure mercury arc lamp. Two modes are observed for species B with each mode splitting into two site absorptions at 2674.3/2667.6 cm^–1^ and 3318.0/3307.2 cm^–1^. These absorptions are observed upon sample deposition, remain almost unchanged upon sample annealing at 12 K, and increase under 280 < *λ* < 580 nm light irradiation but decrease under subsequent 250 < *λ* < 580 nm light irradiation. Two absorptions are observed for species C at 2712.7 and 1420.4 cm^–1^. Both absorptions appear only under 280 < *λ* < 580 nm light irradiation and increase markedly upon 250 < *λ* < 580 nm light irradiation. The experiments were repeated under the same conditions using the ^13^C_2_H_2_, H^12^C^13^CH, H^12^C_2_D, and ^12^C_2_D_2_ samples as well as the natural abundance boron (^10^B: 19.8%; ^11^B: 80.2%) target to aid product identification on the basis of isotopic shifts. The isotopic spectra in selected regions for species C, which is of particular interest here, are shown in Fig. 2 and 3. The product absorptions are summarized in Table 1.

**Table 1:** Experimental and calculated (B3LYP) vibrational frequencies (cm^–1^) and isotope shifts *Δ* (cm^–1^) of the cyclic-HBC_2_BH isotopomers

	^10^B					^11^B	
	C_2_H_2_	^13^C_2_H_2_	*Δ*	C_2_D_2_	*Δ*	C_2_H_2_	*Δ*
Exptl	2712.7	2712.1	0.6	2101.0	611.7	2694.6	18.1
	1420.4	1395.4	25.0	1395.9	24.5	1392.8	27.6
Calcd	2804.8	2804.5	0.3	2133.2	671.6	2789.4	15.4
	1467.0	1439.8	27.2	1393.9	73.1	1436.9	30.1

The group A absorptions are assigned to the cyclic-HBC_2_H radical, which has been reported previously in solid argon.^10*a*^ Two absorptions at 2663.6 and 1146.5 cm^–1^ were assigned to the B–H and B–C_2_ ring stretching modes of the cyclic H^10^BC_2_H molecule in solid argon.^10*a*^ The corresponding H^11^BC_2_H molecule was observed at 2651.4 and 1119.7 cm^–1^ in solid argon.^10*a*^ These two modes are observed at 2708.5 and 1158.2 cm^–1^ for H^10^BC_2_H and at 2692.5 and 1129.1 cm^–1^ for H^11^BC_2_H in the present study in solid neon (Table S1 of the ESI†). Besides these two modes, two additional absorptions at 984.3 and 831.0 cm^–1^ for H^10^BC_2_H and at 978.5 and 820.0 cm^–1^ for H^11^BC_2_H are observed, which can be assigned to the CH bending vibrations.

Species B is tentatively assigned to the inserted HBCCH molecule. Two vibrational modes are observed at 2674.3/2667.6 cm^–1^ and 3318.0/3307.2 cm^–1^ with each mode splitting into two site absorptions. The upper mode shows no boron isotopic shift. The band position and the carbon-13 (3302.9/3292.1 cm^–1^) and deuterium (2673.6/2667.1 cm^–1^) isotopic shifts are indicative of a terminal C–H stretching vibration. The low mode shows almost no carbon isotopic shift. The band position and the boron-11 (2662.2/2655.2 cm^–1^) and deuterium (2060.0/2052.7 cm^–1^) isotopic shifts are appropriate for a B–H stretching vibration (Table S2 of the ESI†). A previous study assigned absorptions at 2084.1 and 2080.4 cm^–1^ to the C–C stretching mode of H^10^BCCH and H^11^BCCH, respectively.^10*a*^

Species C is assigned to a product with B_2_C_2_H_2_ stoichiometry formed from the reaction of two boron atoms with one acetylene molecule based on isotopic substitution experiments. As shown in Fig. 2, the 1420.4 cm^–1^ absorption in the ^10^B-enriched experiment splits into three absorptions at 1420.4, 1407.9 and 1392.8 cm^–1^ in the experiment with a natural abundance boron target. Their relative intensities indicate that this vibration mode involves two equivalent boron atoms. The boron and carbon isotopic shifts (Table 1) indicate that this mode is largely a B–C stretching vibration. The upper absorption at 2712.7 cm^–1^ in the ^10^B-enriched experiment shows a very small shift with ^13^C_2_H_2_ (0.6 cm^–1^) but quite a large shift with ^12^C_2_D_2_ (611.7 cm^–1^). The boron isotopic shift is 18.1 cm^–1^. The band position and isotopic data indicate that the 2712.7 cm^–1^ absorption is a B–H stretching vibration. The spectra for the H^12^C^13^CH, and H^12^C_2_D samples confirm that this species involves two equivalent carbon and hydrogen atoms originating from one C_2_H_2_ reagent. The observation of only one B–H stretching mode and one B–C stretching mode suggests that this B_2_C_2_H_2_ species has high symmetry.

**Fig. 4:**
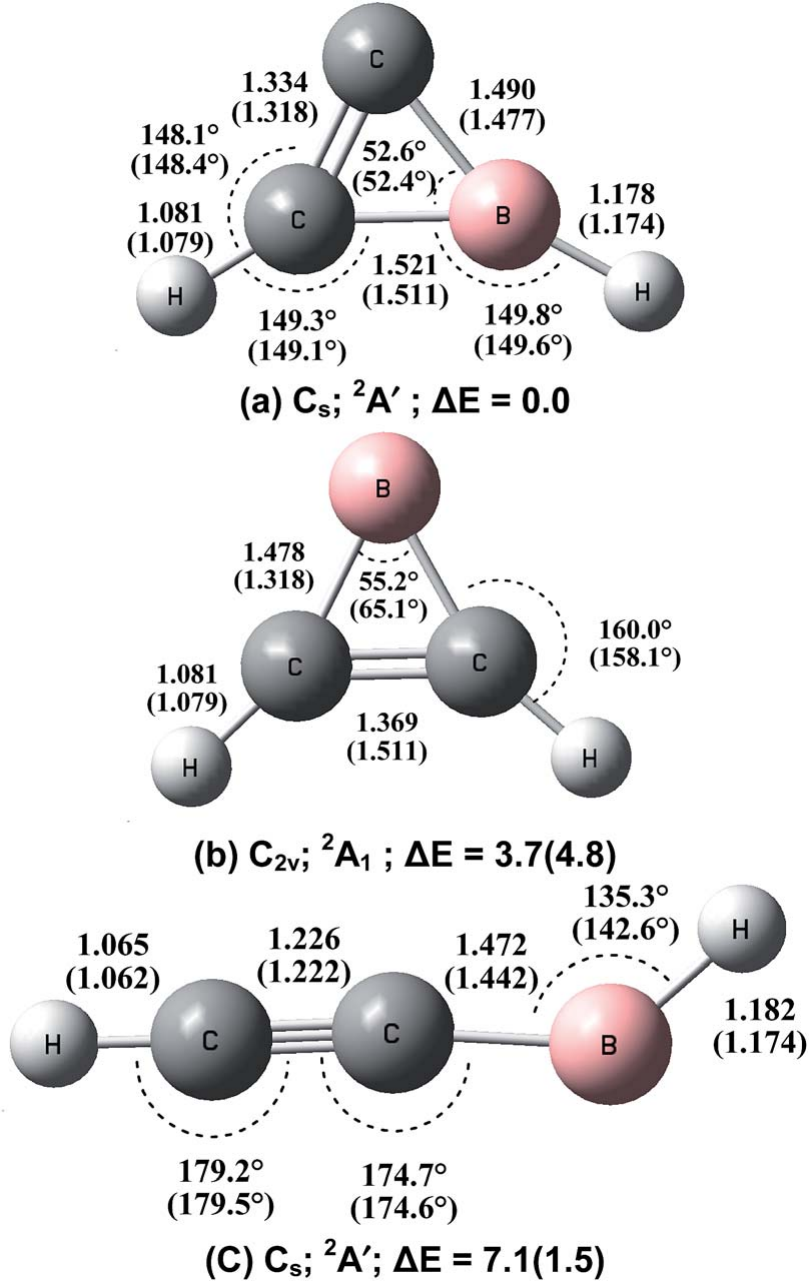
Optimized geometries (bond lengths in angstroms and bond angles in degrees) and relative stabilities (kcal mol^–1^) of the lowest-lying BC_2_H_2_ isomers at the CCSD(T)/aug-cc-pVTZ and B3LYP/aug-cc-pVTZ (in parentheses) levels.

In order to verify the experimental assignments, quantum chemical calculations were performed at both the B3LYP and CCSD(T) levels of theory. Calculations were first performed on various possible structural isomers of the 1 : 1 BC_2_H_2_ species at the B3LYP level (Fig. S1 of the ESI†). The three lowest-lying structures were re-optimized at the CCSD(T) level, and the optimized geometries at both levels of theory are shown in Fig. 4. In agreement with previous studies,^5,6^ the most stable structure is the cyclic-HBC_2_H radical, which has a ^2^A′ ground state with planar *C*_s_ symmetry. The B–H and B–C_2_ stretching modes are predicted at the B3LYP level to be the most intense absorptions with isotopic shifts in good agreement with the experimental values (Table S1†). The inserted HBCCH isomer is predicted to be bent with a HBC bond angle of 135.3° at the CCSD(T) level or 142.6° at the B3LYP level. This structure is 1.5 kcal mol^–1^ (B3LYP) or 7.1 kcal mol^–1^ (CCSD(T)) less stable than the most stable cyclic-HBC_2_H isomer. As listed in Table S2,† the C–H and B–H stretching modes of the ^2^A′ ground state H^11^BCCH molecule are calculated to be 3449.7 and 2768.7 cm^–1^ at the B3LYP level. The CCSD(T) calculations give slightly lower values of 3447.2 and 2731.4 cm^–1^, which are in quite good agreement with the experimental values in solid neon. These two modes are predicted to be the most intense vibrations (72 and 54 km mol^–1^ at the B3LYP level). The C–C stretching mode is predicted at 2002.8 cm^–1^ with a very low IR intensity (10 km mol^–1^) at the B3LYP level. Besides the cyclic-HBC_2_H and inserted HBCCH isomers, previous argon matrix experiments have reported the formation of a third BC_2_H_2_ isomer, namely, the cyclic B(C_2_H_2_) borirene radical,^10^ which is not observed in the present study in solid neon. The borirene radical is predicted to be only 3.7 kcal mol^–1^ less stable than the global minimum structure at the CCSD(T) level (Fig. 4). As has been discussed previously,^6*c*^ the B + C_2_H_2_ reaction was predicted to proceed with the initial formation of the cyclic-B(C_2_H_2_) borirene radical, which can further isomerize to the cyclic-HBC_2_H and HBCCH isomers. Note that the B + C_2_H_2_ addition reaction to form the borirene radical is highly exothermic and that the barriers of the subsequent isomerization reactions to form the cyclic-HBC_2_H and inserted HBCCH isomers lie much lower in energy than the reactants B + C_2_H_2_.^6*c*^ Therefore, the stabilization of the borirene radical may not be possible in the more inert neon matrix if the reaction energy cannot be relaxed efficiently. A recent study on the B + C_2_H_4_ reaction found that the ground state boron atom inserts spontaneously into the C=C double bond of C_2_H_4_ in forming the allene-like H_2_CBCH_2_ molecule in solid neon.^12^ Although the addition reaction species cyclic-B(C_2_H_4_) is predicted to be a stable intermediate on the potential energy surface in forming the inserted H_2_CBCH_2_ isomer, this cyclic-B(C_2_H_4_) intermediate was not observed experimentally.

**Fig. 5:**
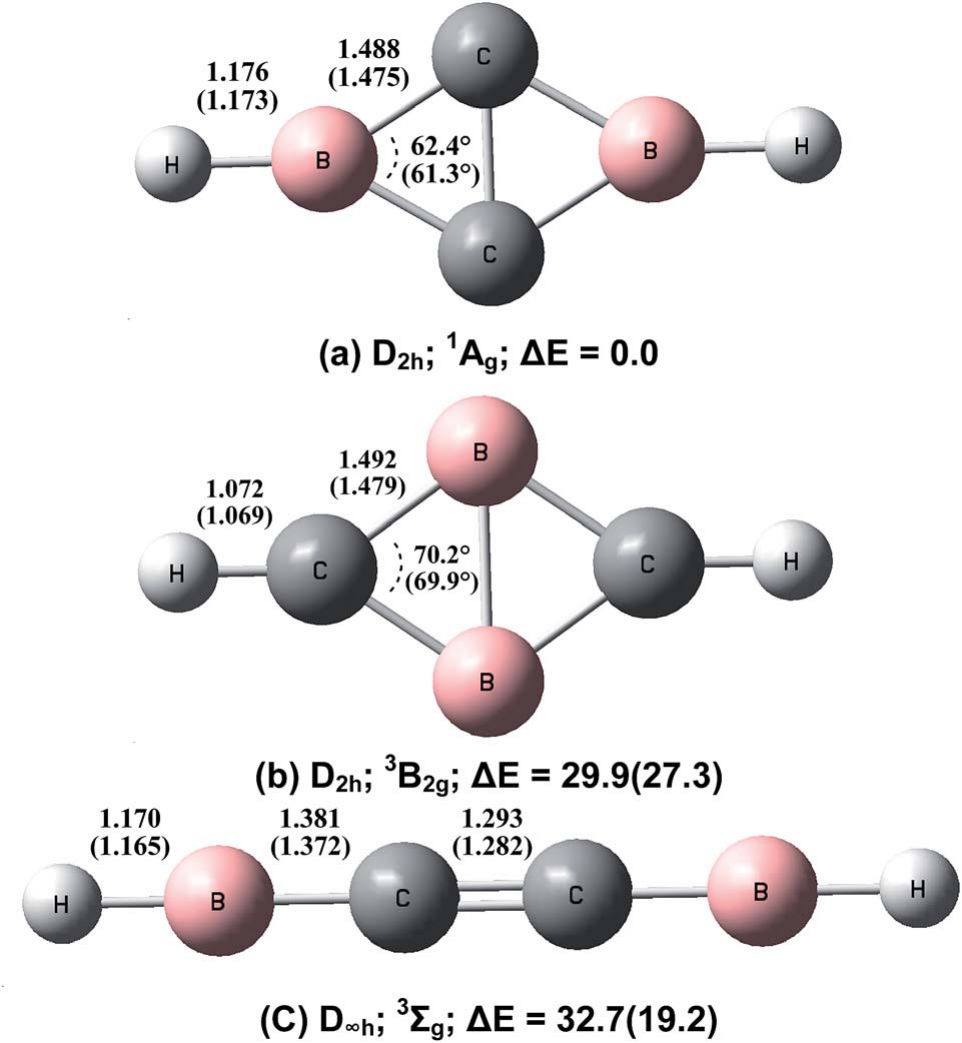
Optimized geometries (bond lengths in angstroms and bond angles in degrees) and relative stabilities (kcal mol^–1^) of the B_2_C_2_H_2_ isomers at the CCSD(T)/aug-cc-pVTZ and B3LYP/aug-cc-pVTZ (in parentheses) levels.

Geometric optimizations were also performed on various possible structures of B_2_C_2_H_2_ involving equivalent boron, carbon and hydrogen atoms. The optimized structures and relative stabilities are shown in Fig. 5. At both levels of theory, the global energy minimum structure of B_2_C_2_H_2_ is a cyclic-HBC_2_BH molecule involving a rhombic B_2_C_2_ ring and two terminal B–H bonds (structure (a)). This structure has a closed-shell singlet (^1^A_g_) ground state with planar *D*_2h_ symmetry. The four C–B bonds are equivalent with a bond length of 1.488 Å at the CCSD(T) level, shorter than the sum of the single bond covalent radii of boron and carbon (1.60 Å), but longer than the sum of their double-bond covalent radii (1.45 Å).^28^ The C–C bond distance is 1.54 Å, which indicates the existence of a bonding interaction. The second structure (b) also involves a rhombic B_2_C_2_ ring but the two hydrogen atoms are bonded upon the carbon atoms. This structure has a triplet (^3^B_2g_) ground state with planar *D*_2h_ symmetry. The predicted B–C bond length of 1.492 Å at the CCSD(T) level is slightly longer than that in structure (a). The B–B bond distance is 1.715 Å, close to a single bond value.^29^ Population analysis indicates that the two unpaired spins are almost evenly distributed over the rhombic ring. This isomer is predicted to be 29.9 (CCSD(T)) or 27.3 (B3LYP) kcal mol^–1^ higher in energy than the global minimum structure. The third isomer has a triplet ground state with a linear HBCCBH structure. At the CCSD(T) level, the C–C bond length is 1.293 Å, close to a typical C=C double bond.^28^ The two B–C bond distances are predicted to be 1.381 Å, slightly shorter than typical B–C double bonds.^28^ It can roughly be regarded as an electron-deficient cumulene. This structure is 32.7 kcal mol^–1^ (CCSD(T)) or 19.2 kcal mol^–1^ (B3LYP) less stable than the most stable structure (a).

The identification of the cyclic-HBC_2_BH species is achieved by comparing the experimental data with the calculated harmonic frequencies and the isotope shifts (*Δ*) of the global energy minimum structure at the B3LYP level, which are shown in Table 1. The vibrational frequencies calculated at the CCSD(T) level are very close to the B3LYP values (Table S4†); therefore, only the calculated values at the B3LYP level will be discussed. Table 1 shows that the absolute values of the B–H and B–C stretching modes of the cyclic-H^10^BC_2_^10^BH molecule predicted at 2804.8 and 1467.0 cm^–1^ are slightly higher than the experimentally observed values. It is well known that the calculated harmonic frequencies are, in general, higher than the experimental anharmonic frequencies.^30^ The noble-gas matrix effect, which is expected to be quite small for neon matrix,^31^ is another factor that contributes to the difference between the matrix experimental and computed values. These two modes are predicted to be the most intense IR absorptions (Table S4†). The calculated isotopic shifts *Δ* of the two modes agree very well with the experimental values, confirming unequivocally that the experimentally observed vibrations are due to the cyclic-HBC_2_BH structure, which is also predicted to be the global energy minimum structure of B_2_C_2_H_2_.

**Fig. 6:**
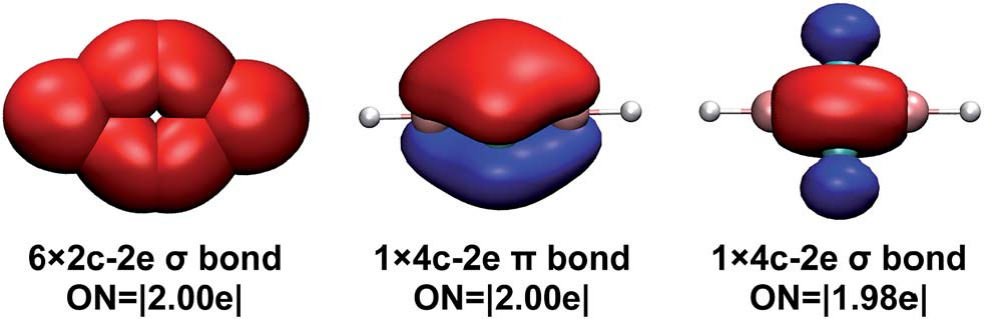
Chemical bonding pattern of cyclic-HBC_2_BH shown by adaptive natural density partitioning analysis (AdNDP). ON denotes occupation number.

To understand the bonding in the cyclic-HBC_2_BH molecule, we performed Adaptive Natural Density Partitioning (AdNDP) analysis, which has the ability to recover simultaneously both localized and delocalized bonding in chemical species.^32^ The results for the cyclic-HBC_2_BH molecule are shown in Fig. 6. Besides six 2c–2e localized σ bonds (four C–B and two B–H σ bonds), the AdNDP analysis reveals one delocalized 4c–2e σ bond and one delocalized 4c–2e π bond for the central cyclic-B_2_C_2_ moiety. Both the delocalized σ and π electrons satisfy the 4*N* + 2 rule for aromaticity with *n* = 0. Thus, the cyclic-HBC_2_BH molecule is doubly (σ and π) aromatic. The bonding of the cyclic-B_2_C_2_ moiety is about the same as that of B_4_, which was characterized to have a rhombus global minimum structure that is doubly aromatic as well.^33^ The aromaticity of cyclic-HBC_2_BH is further supported by the widely used nuclear independent chemical shift (NICS) calculations, which were suggested as reliable indicators of aromaticity in non-fused cyclic systems.^27^ The NICS value at 1 Å above the ring center is –22.2. The negative NICS value indicates that the cyclic-HBC_2_BH molecule possesses significant aromatic character. The corresponding value for the borirene radical involving a three-membered BC_2_ ring, which has been characterized as a 2π electron aromatic species, is –14.0. The cyclic-HBC_2_H radical is predicted to have a NICS(1) value of –12.6, suggesting that it is also an aromatic species (Table 2).

**Table 2:** Calculated NICS values at and 1 Å above the ring centers of the cyclic-HBC_2_BH, HBC_2_H and B(C_2_H_2_) species

	NICS(0)	NICS(1)
HBC_2_BH	–29.6	–22.2
HBC_2_H	–35.1	–12.6
B(C_2_H_2_)	–27.0	–14.0

The experimental observations clearly show that the cyclic-HBC_2_BH molecule is formed *via* reactions of two boron atoms and acetylene in solid neon, demonstrating that both C–H bonds of acetylene can be activated by atomic boron. The formation of an aromatic cyclic-HBC_2_BH molecule from two boron atoms and acetylene is predicted to be highly exothermic by 206.7 kcal mol^–1^ at the CCSD(T) level. The activation of the C–H bond is one of the most important processes in organic synthetic chemistry.^34^ Although many transition metal complexes were found to be able to activate a single C–H bond^35,36^ or even multiple C–H bonds^37^ of alkanes, alkenes and their derivatives, the activation of the C–H bond of acetylene still remains an important challenge. The C–H bond of acetylene is one of the strongest known bonds with the bond dissociation energy even larger than that of the C–H bonds of methane and ethylene.^34^ The present finding reveals the very first example of metal-free double C–H bond activation of acetylene.^38^ It provides a possible new reaction route for metal-free C–H bond activation of acetylene in forming new aromatic organo-boron compounds.

## Conclusion

The reactions of boron atoms with acetylene are investigated using matrix isolation infrared spectroscopy in solid neon. The species formed are identified *via* isotopic substitutions as well as quantum chemical calculations. Besides the previously reported single C–H bond activation species cyclic-HBC_2_H and inserted HBCCH molecules, a new cyclic-HBC_2_BH diboron species is also formed under UV-visible light excitation, demonstrating that both C–H bonds of acetylene can be activated by atomic boron. The cyclic-HBC_2_BH molecule is characterized as having a closed-shell singlet ground state with planar *D*_2h_ symmetry that is doubly aromatic. The present finding reveals the very first example of double C–H bond activation of acetylene. This provides a possible new reaction route for metal-free C–H bond activation of acetylene in forming new organo-boron compounds.
