# Supplementary material for: Double C–H bond activation of acetylene by atomic boron in forming aromatic cyclic-HBC2BH in solid neon
Source: Chem Sci. 2017 Apr 19;8(6):4443–9. doi: 10.1039/c7sc01399j (PMC5590098; doi:10.1039/c7sc01399j)
Supplement: Supplementary file 1 [file SC-008-C7SC01399J-s001.pdf]

Electronic Supplementary Material (ESI) for Chemical Science.
This journal is © The Royal Society of Chemistry 2017

Supporting Information

Double C-H bond activation of acetylene by atomic boron in forming aromatic cyclic-HBC_2BH in solid neon

Jiwen Jian, Wei Li, Xuan Wu, Mingfei Zhou*

Department of Chemistry, Shanghai Key Laboratory of Molecular Catalysis and Innovative Materials & Collaborative Innovation Center of Chemistry for Energy Materials, Fudan University, Shanghai 200433, China. E-mail: mfzhou@fudan.edu.cn

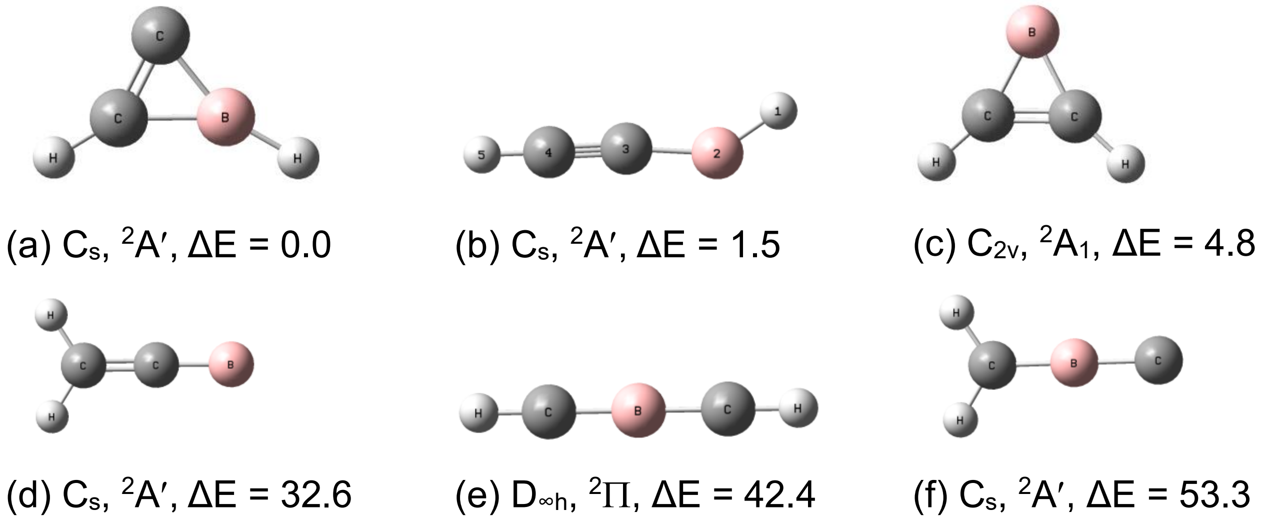

Figure S1. Optimized geometries (bond lengths in angstroms and bond angles in degrees) and relative stabilities (kcal/mol) of the BC_2H_2 isomers at the B3LYP/aug-cc-pVTZ level.

Table S1. Experimental and calculated vibrational frequencies (cm^{-1}) and intensities (km/mol in parentheses) of the cyclic-HBC_2H isotopomers.

$H^{10}B^{12}C_2H$		$H^{10}B^{13}C_2H$		$D^{10}B^{12}C_2D$		$H^{11}B^{12}C_2H$		
B3LYP	Exptl.	B3LYP	Exptl.	B3LYP	Exptl.	B3LYP	Exptl.	CCSD(T)
3216.1(0.3)		3203.8(0)		2408.9(1)		3216.0(0.3)		3232.3
2793.1(56)	2708.5	2792.9(56)	2708.5	2109.1(54)	2066.4	2778.1(52)	2692.5	2776.9
1599.8(3)		1543.7(3)		1539.8(4)		1596.7(3)		1554.9
1185.9(69)	1158.2	1168.4(66)	1141.2	1133.4(53)	1098.2	1155.7(67)	1129.1	1143.9
1007.2(28)	984.3	984.9(30)	963.0	959.6(7)		1001.1(30)	978.5	1000.0
882.4(45)	831.0	876.9(30)	827.1	717.7(26)		876.3(45)	820.0	869.2
849.1(25)		844.5(23)		685.5(20)		836.8(23)		840.9
736.4(13)		732.8(12)		543.4(11)		735.7(12)		743.2
732.6(2)		729.9(2)		567.1(0)		729.1(1)		717.0

Table S2. Experimental and calculated vibrational frequencies (cm^{-1}) and intensities (km/mol in parentheses) of the HBCCH isotopomers.

$H^{10}B^{12}C_2H$		$H^{10}B^{13}C_2H$		$D^{10}B^{12}C_2D$		$H^{11}B^{12}C_2H$		
B3LYP	Exptl.	B3LYP	Exptl.	B3LYP	Exptl.	B3LYP	Exptl.	CCSD(T)
3449.7(72)	3318.0<br>3307.2	3433.2(71)	3302.9<br>3292.1	2639.6(34)	2673.6<br>2667.1	3449.7(72)	3318.0<br>3307.2	3447.2
2782.4(54)	2674.3<br>2667.6	2782.2(53)	2674.3<br>2667.6	2095.0(53)	2060.0<br>2052.7	2768.7(51)	2662.2<br>2655.2	2731.4
2002.8(10)		1931.1(9)		1883.5(6)		2001.9(10)		2060.3
1064.5(20)		1052.1(20)		1011.8(10)		1035.3(20)		988.6
797.8(22)		789.8(22)		636.1(10)		797.8(22)		768.0
539.2(35)		535.4(35)		473.1(16)		532.1(34)		680.2
431.1(61)		427.6(62)		342.7(23)		431.1(61)		515.6
340.9(14)		332.9(11)		278.3(22)		340.5(14)		323.1
273.8(5)		266.3(4)		253.7(3)		273.5(5)		249.9

Table S3. Calculated vibrational frequencies (cm^{-1}) and intensities (km/mol in parentheses) of the cyclic-B(C_2H_2) isotopomers.

$^{10}B(^{12}C_2H_2)$	$^{10}B(^{13}C_2H_2)$	$^{10}B(^{12}C_2D_2)$	$^{11}B(^{12}C_2H_2)$	
B3LYP	B3LYP	B3LYP	B3LYP	CCSD(T)
3221.7(1)	3208.6(1)	2425.5(0)	3221.5(1)	3235.7
3201.8(4)	3191.4(4)	2366.2(2)	3201.8(4)	3214.7
1540.1(1)	1498.0(2)	1466.1(1)	1526.5(0)	1490.4
1234.6(74)	1209.0(70)	1232.1(68)	1206.1(73)	1186.9
1187.6(5)	1160.1(6)	1115.1(0)	1179.8(6)	1170.0
1024.1(1)	1012.8(0)	821.2(0)	1024.1(0)	1002.0
928.1(26)	920.7(26)	759.0(15)	917.1(26)	906.9
887.7(20)	887.3(20)	638.2(12)	886.4(19)	888.3
754.3(45)	749.6(45)	587.0(21)	752.6(45)	729.1

Table S4. Calculated vibrational frequencies (cm^{-1}) and intensities (km/mol in parentheses) of the cyclic-HBC_2BH isotopomers.

$H^{10}B^{12}C_2{}^{10}BH$	$H^{10}B^{13}C_2{}^{10}BH$	$D^{10}B^{12}C_2{}^{10}BD$	$H^{11}B^{12}C_2{}^{11}BH$	
B3LYP	B3LYP	B3LYP	B3LYP	CCSD(T)
2809.5(0)	2809.4(0)	2131.4(0)	2793.6(0)	2794.0
2804.8(176)	2804.5(174)	2133.2(237)	2789.4(160)	2790.1
1467.0(314)	1439.8(303)	1393.9(226)	1436.9(305)	1413.7
1269.4(0)	1252.2(0)	1235.8(0)	1237.9(0)	1210.5
1237.4(0)	1201.7(0)	1210.3(0)	1223.2(0)	1203.8
892.2(33)	889.8(31)	787.3(68)	880.4(28)	888.4
866.2(0)	865.2(0)	837.0(0)	858.5(0)	859.7
855.9(0)	833.6(0)	674.2(0)	841.8(0)	795.6
800.1(52)	797.5(52)	678.3(40)	788.6(50)	773.6
772.1(0)	772.1(0)	615.8(0)	762.5(0)	762.8
666.0(46)	655.3(45)	545.4(10)	657.4(47)	665.0
350.1(1)	344.7(1)	298.4(0)	346.0(1)	334.1

Table S5. Calculated vibrational frequencies (cm^{-1}) and intensities (km/mol in parentheses) of the cyclic-HCB_2CH isotopomers.

$H^{12}C^{10}B_2{}^{12}CH$	$H^{13}C^{10}B_2{}^{13}CH$	$D^{12}C^{10}B_2{}^{12}CD$	$H^{12}C^{11}B_2{}^{12}CH$	
B3LYP	B3LYP	B3LYP	B3LYP	CCSD(T)
3332.1(0)	3320.0(0)	2478.4(0)	3332.0(0)	3330.9
3328.6(17)	3317.3(17)	2465.4(9)	3328.5(17)	3328.9
1290.7(0)	1270.9(0)	1259.3(0)	1257.3(0)	1241.7
1230.7(0)	1209.7(0)	1198.0(0)	1204.9(0)	1182.0
1051.2(0)	1049.5(0)	992.6(0)	1020.5(0)	1012.5
923.0(0)	905.4(0)	901.8(0)	899.2(0)	900.3
908.1(23)	903.9(25)	758.3(4)	907.7(24)	889.6
786.7(0)	775.3(0)	638.5(0)	781.9(0)	760.9
721.1(33)	711.0(31)	624.1(33)	702.7(31)	700.0
593.6(0)	588.3(0)	465.4(0)	593.6(0)	560.3
536.6(88)	532.3(86)	503.2(30)	535.3(87)	517.6
487.6(0)	482.4(1)	375.8(26)	476.2(0.2)	463.2

Table S6. Calculated vibrational frequencies (cm^{-1}) and intensities (km/mol in parentheses) of the linear HBCCBH isotopomers.

$H^{10}B^{12}C^{12}C^{10}BH$	$H^{10}B^{13}C^{13}C^{10}BH$	$D^{10}B^{12}C^{12}C^{10}BD$	$H^{11}B^{12}C^{12}C^{11}BH$	
B3LYP	B3LYP	B3LYP	B3LYP	CCSD(T)
2886.1(0)	2885.2(0)	2256.0(0)	2868.7(0)	2851.3
2884.0(37)	2883.5(36)	2209.4(51)	2866.8(33)	2849.6
2041.3(0)	1970.4(0)	1950.4(0)	2034.4(0)	2012.6
1530.1(62)	1501.8(60)	1443.4(43)	1499.4(60)）	1476.9
946.4(0)	942.1(0)	896.3(0)	911.1(0)	894.0
610.4(6)	609.4(3)	493.9(10)	603.5(6)	599.2
609.7(0)	608.5(0)	512.1(0)	602.8(0)	594.9
444.9(0)	429.8(0)	407.3(0)	444.4(0)	417.6
193.8(15)	190.5(14)	173.1(6)	191.0(15)	183.6
